# Supplementary material for: Influence of Interpenetrating Chains on Rigid Domain Dimensions in Siloxane-Based Block-Copolymers
Source: Polymers (Basel). 2022 Sep 27;14(19):4048. doi: 10.3390/polym14194048 (PMC9572696; doi:10.3390/polym14194048)
Supplement: Supplementary file 1 [file polymers-14-04048-s001.zip › polymers-1927738-supplementary.pdf]

The description of NMR experiments

The NMR measurements were carried out on a Bruker Avance II 500 spectrometer operating at a proton frequency of 500.13 MHz 4 mm double resonance MAS probe head was used for recording all spectra.

(1) DQ build-up and spin-diffusion experiments. The method used for ^{1}H DQ spin-diffusion measurements was the same as we used in our previous work [1]. The spectral width was 200 ppm (100 kHz) to cover the broad hard-phase (HS) signal. Pulse sequence is a conventional 5-pulse DQ experiment with a variable z-filter with proper phase cycling for DQ filtration. DQ build-up curves were measured using the same scheme by varying excitation/reconversion time with additional 180° pulses in excitation and reconversion segments to compensate for spectral offsets and chemical-shift dispersion at long evolution times. For spin-diffusion experiments, the DQ excitation and reconversion time τ_{DQ} of 15 μs was used to obtain maximum hard-phase signal with negligible inter- and mobile-phase magnetization, because it corresponds to the maxima of the DQ build-up curves at short times (the range of the strong ^{1}H dipolar interactions). For the separate integration of individual resolved mobile signals a narrow 20 ppm region around the sharp signals was baseline-corrected using a second order polynomial, thus removing the HS background hump. These integrations yield the overall mobile fraction (SS). All spin diffusion experiments in this work were performed under static conditions to avoid any influence of MAS on the spin diffusion coefficients.

(2) ^{1}H Hahn echo NMR experiment was employed to measure the transverse magnetization relaxation (T_2 decay). Additional experiments with the dipolar filter before echo sequence were performed to obtain the T_2 of the mobile component directly, which could be used to determine the proton spin diffusion coefficient for the mobile polymers as proposed by Mellinger et al. [2] The experimental conditions of the dipolar filter sequence before Hahn echo were the same as used in corresponding ^{1}H dipolar filter experiments [1].

(3) CP-MAS experiments. Conventional cross-polarization MAS NMR experiment (CP-MAS) with high power proton decoupling (100 kHz) was used to detect the signals from rigid component with high CP efficiency, recycle delay was 5 s, CP contact time - 2 ms.

(4) ^{13}C direct-polarization MAS NMR experiment with echo detection (90˚-180˚-aq) and low power proton decoupling (LPD, 25 kHz) was used to observe the mobile component. Applying 180˚ pulse after delay refocuses mobile component whereas the broad signals from the rigid part are already irreversibly relaxed. Recycle delay in this experiment was 0.5 s (optimal for short relaxing of mobile component), echo delay - 3ms. Spinning frequency in all experiments was 5-8 kHz. Spectra were recorded at ambient temperature.

(5) For 2D EXSY experiments, π/2 pulse duration was 2.5 μs, 4 s recycle delay was used. 25 and 50 ms mixing times were used to examine spin diffusion.

[1] Mokeev, M.V.; Zuev, V.V. Rigid phase domain sizes determination for poly(urethane-urea)s by solid-state NMR spectroscopy. Correlation with mechanical properties. *Eur. Polym. J.* **2015**, *71*,372-379. https://doi.org/10.1016/j.eurpolymj.2015.08.003

[2] Mellinger, F.; Wilhelm, M.; Spiess, H.W. Calibration of ^{1}H NMR Spin Diffusion Coefficients for Mobile Polymers through Transverse Relaxation Measurements. *Macromolecules*, **1999**, *32 (14)*, 4686-4691. https://doi.org/10.1021/ma9820265

Calculation of domain size using spin-diffusion experiment

We using next equation [1]:

$$d_{dis}=\frac{(\rho_A^H\varphi_A+\rho_B^H\varphi_B)}{\varphi_A\varphi_B}\times\frac{4\varepsilon\varphi_B}{\sqrt{\pi}}\times\frac{\sqrt{D_AD_B}}{\rho_A^H\sqrt{D_A}+\rho_B^H\sqrt{D_B}}\times\sqrt{t_m^{s,0}} \quad (1)$$

Here φ_m (m = A, B) are volume fractions of the mobile phase A and rigid phase B, ρ_A^H and ρ_B^H are proton densities, and D_A and D_B are spin-diffusion coefficients of mobile and rigid phases. The ε represents dimensionality, its value depends on the morphology and is equal 2 for cylindrical form of hard domains [1]. $t_m^{s,0}$ is the characteristic mixing time of spin-diffusion introduced by Mellinger et al. [2], which can be determined from the intercept of extrapolated initial linear-slope with the X-axis.

The diffusion coefficient of the rigid phase (D_B) can be calculated from the following equation that is valid for the Gaussian line shape [3]:

$$D_B=\frac{1}{12}\sqrt{\frac{\pi}{2\ln 2}}<r^2>\Delta\nu_{1/2}^B \quad (2)$$

Here $<r^2>$ is the mean-square distance between the nearest spins (we use a value of 0.05 nm^2) [4], $\Delta\nu_{1/2}^B$ is the full width at half height for proton signals of rigid phase obtained by solid-state ^{1}H NMR experiment with a double-quantum (DQ) filter. The spin-diffusion coefficients for mobile phase D_A were calculated using T_2 measurements of mobile fraction (from dipolar filtered Hahn-echo experiment) by method proposed earlier [5] The characteristic size (L) of the polymer matrix, namely the distance between the centers of two closest neighboring dispersed HS domains, i.e. long period, is calculated using equation:

$$L = \frac{d_{dis}}{\sqrt[\varepsilon]{1 - \varphi_B}} \quad (3)$$

1. Clauss J, Schmidt-Rohr K, Spiess HW. (1993) Determination of domain sizes in heterogeneous polymers by solid-state NMR. Acta Polymerica 44(1): 1-17. DOI: 10.1002/actp.1993.010440101
2. Mellinger F, Wilhelm M, Spiess HW. (1999) Calibration of ^{1}H NMR Spin Diffusion Coefficients for Mobile Polymers through Transverse Relaxation Measurements. Macromolecules 32 (14):4686-4691. https://doi.org/10.1021/ma9820265
3. Shu J, Li P, Chen Q, Zhang S. (2010) Quantitative measurement of polymer compositions by NMR spectroscopy: targeting polymers with marked difference in phase mobility. Macromolecules 43(22): 8993-8999. https://doi.org/10.1021/ma101711f
4. Voda A, Beck K, Schauber T, Adler M, Dabisch T, Bescher M, Viol M, Demco D, Blumich B. (2006) Investigation of soft segments of thermoplastic polyurethane by NMR, differential scanning calorimetry and rebound resilience. Polymer Testing 25(2): 203–213. 10.1016/j.polymertesting.2005.10.007
5. Li XJ, Fu WG, Wang YN, Chen TH, Liu XH, Lin H, Sun P, Jin Q, Ding D. (2008) Solid-State NMR characterization of unsaturated polyester thermoset blends. Polymer 49: 2886-2897. 10.1016/j.polymer.2008.04.042
